# Cigarette smoke extract exposure induces differential morphological and transcriptional responses in the ovary and testes of mice

**DOI:** 10.3389/ftox.2026.1943043

**Published:** 2026-10-14

**Authors:** Yasmin Azizbayli, Laura G. Bartlett, Amanda Tatler, Victoria James, Marcos Castellanos-Uribe, Iqbal Khan, Nazia Nazar, Sean T. May, Adam J. Watkins, Lucy C. Fairclough

**Affiliations:** 1 School of Life Sciences, The University of Nottingham, Nottingham, United Kingdom; 2 School of Medicine, The University of Nottingham, Nottingham, United Kingdom; 3 School of Veterinary Medicine and Science, Biodiscovery Institute, University of Nottingham, Nottingham, United Kingdom; 4 Nottingham Arabidopsis Stock Centre (NASC), School of Biosciences, University of Nottingham, Loughborough, United Kingdom; 5 Division of Clinical Medicine, School of Medicine and Population Health, Beech Hill Road, University of Sheffield, Sheffield, United Kingdom

**Keywords:** cigarette smoke, histological evaluation, intranasal exposure, murine model, reproductive organs, transcriptomic analysis

## Abstract

**Introduction:**

Cigarette smoking is a major public health concern and is associated with impaired reproductive function in both females and males. While smoking related fertility defects have been reported, the molecular mechanisms underlying smoke-induced reproductive toxicity, particularly in gonadal tissues, remain poorly defined.

**Methods:**

An in vivo mouse model of cigarette smoke extract exposure was utilized. Cigarette smoke was drawn through RPMI medium and diluted to create a light-dose (1%, representing a light smoker), or heavy-dose (3%, representing a heavy smoker) cigarette smoke extract. Sixteen eight-week-old male and female C57BL/6J mice were sedated with isoflurane and administered either the 1%, 3% or control (RPMI medium alone) intranasally three times per week for 12 weeks. After 12 weeks, ovarian and testicular tissues were collected for either histological analysis of tissue morphology (n = 4 mice per male/female gonad) or for transcriptomic analysis by microarray (n = 4 mice per male/female gonad). No detrimental impact of our cigarette smoke extracts on animal welfare was observed.

**Results:**

While there was no measurable change in ovarian histology between groups, significant downregulation of transcription factors and genes regulating cell division in response to the 1% and 3% extract was observed. In addition, increased expression of multiple ribosomal proteins in both the 1% and 3% exposure females was also observed. In males, a significant increase in mean seminiferous tubule cross-section area in 3% males was observed.

**Discussion:**

Transcriptomic analysis identified differential expression of genes associated with immune responses, spermatogenesis and male fertility across the groups. Our data highlight the significant impacts of cigarette smoking on reproductive health in males and females. Our data also highlights the differential responses of ovarian and testicular tissue to the effects of smoking, suggesting parental specific influences of smoking on reproductive health, fertility and potentially offspring wellbeing.

## 1 Introduction

Active and passive smoking have negative effects on health and can contribute to diabetes, heart disease, and many cancers. In the United Kingdom, cigarette consumption among adults has declined over time (Jackson et al., 2025). Nevertheless, tobacco use remains a major global public-health concern. The World Health Organization estimated that approximately 1.25 billion adults used tobacco in 2022 (WHO, 2024). Cigarettes vary globally in composition, quality, price, and regional availability. Cigarette smoke contains a complex mixture of chemicals, including nicotine, carbon monoxide, polycyclic aromatic hydrocarbons, and toxic metals such as lead and cadmium. Several smoke constituents can disrupt endocrine signalling, increase oxidative stress, and damage reproductive cells and tissues (Dechanet et al., 2011). Tobacco-smoke exposure is therefore an important, modifiable risk factor for adverse reproductive outcomes. However, the molecular pathways through which cigarette smoke affects the gonads remain incompletely defined.

In males, smoking is associated with reduced semen quality, reproductive endocrine dysfunction, and impaired spermatogenesis, sperm maturation, and sperm function (Osadchuk et al., 2023). Tobacco smoke has also been linked to oxidative stress, cellular damage, and apoptosis within the testis and sperm, which may collectively affect male fertility (Viloria et al., 2010). Smoking may also affect reproductive hormones, although reported patterns are inconsistent. Some studies have reported changes in luteinising hormone, prolactin, and testosterone without corresponding changes in follicle-stimulating hormone, estradiol, or inhibin B (Dai et al., 2015). Higher total and free testosterone levels have also been associated with greater daily cigarette consumption (Svartberg and Jorde, 2007). Conversely, other studies have reported lower testosterone levels or no significant hormonal changes (Bundhun et al., 2019). These findings should not be interpreted as evidence of a beneficial effect of smoking, as their biological significance remains uncertain. Studies of sperm DNA have more consistently reported increased chromatin decondensation and DNA fragmentation in smokers (Aboulmaouahib et al., 2018). Infertile smokers have also shown increased sperm DNA fragmentation and apoptotic markers, together with poorer motility, morphology, and semen pH (Ranganathan et al., 2019). In mice, cigarette-smoke exposure increased single- and double-stranded DNA breaks in testicular tissue and spermatozoa (La Maestra et al., 2015). Collectively, these findings indicate that cigarette smoke may affect male reproductive tissues through interacting endocrine, oxidative, and cellular mechanisms.

In females, smoke components can affect several reproductive cell types, including oocytes, granulosa cells, luteal cells, and endothelial cells (Lee et al., 2017). The Norwegian Mother and Child Cohort Study (MoBa) is a large prospective cohort used to evaluate prenatal exposures and later health outcomes (Magnus et al., 2006). Prenatal tobacco exposure has also been associated with altered infant stress responses (Stroud et al., 2014). Human evidence suggests possible longer-term reproductive consequences, including reduced later fecundability among adult women exposed to tobacco smoke *in utero* (Ye et al., 2010). Placental transcriptomic analysis has identified altered expression of genes involved in xenobiotic metabolism, including CYP1A1, CYP1B1, CYB5A, and COX4I2, in smokers compared with non-smokers (Bruchova et al., 2010). Cigarette-smoke exposure has additionally been associated with downregulation of placental steroid-metabolising enzymes, including CYP19A1 and HSD3B1 (Bruchova et al., 2010). Together, these findings indicate that tobacco smoke can disrupt placental and endocrine function, although the molecular responses within ovarian tissue remain incompletely defined.

Animal models are widely used to investigate the reproductive effects of cigarette smoke. Common approaches include whole-body and nose-only inhalation systems (Kogel et al., 2021). However, these methods have important welfare and methodological limitations. Comparisons between whole-body and nose-only exposure have identified stronger inflammatory responses following whole-body exposure (Serré et al., 2021). This difference may partly reflect ingestion of particulate matter deposited on the fur during grooming (Gue et al., 2004). Transdermal absorption has also been proposed, supported by higher serum cotinine concentrations in whole-body-exposed animals (Xu et al., 2014). These limitations support the development of controlled alternative exposure methods that reduce restraint-related stress and other confounding effects.

In this exploratory study, we used our previously published intranasal cigarette-smoke extract model (Bartlett et al., 2026) to investigate exposure-associated morphological and transcriptional changes in the ovary and testis. Unlike our previous publication, which focused on peripheral immune responses and lung pathology, the present analysis examines gonadal histology and tissue-specific microarray profiles following exposure to control, 1%, or 3% cigarette-smoke extract. We aimed to identify differential ovarian and testicular responses and generate hypotheses concerning pathways that may contribute to smoke-associated reproductive toxicity. These analyses were intended to identify patterns for future functional validation rather than to demonstrate fertility impairment directly.

## 2 Methods

### 2.1 Cigarette smoke extract preparation

To generate our cigarette smoke extract (CSE), a custom smoke-collection apparatus consisting of plastic piping connected to a three-way stopcock was used (See Appendix A). The stopcock was attached to one side to a 50 mL BD Plastipak polypropylene disposable Luer-Lok concentric-tip syringe (Cat. No. SYR6168, Scientific Laboratory Supplies, United Kingdom) while the other was connected to a modified 25 mL tube lid, allowing attachment of the CSE collection tube. Marlboro Red cigarettes were used as the standardised cigarette and were secured using 1 mL pipette tips with the conical ends removed. The cigarette smoke was drawn through phenol-red–free RPMI-1640 culture medium (Cat no. R6504, Merck, United Kingdom) containing 2 mM L-glutamine (Cat. No. R8755, Merck, United Kingdom) prior to sterile filtration (Sartorius Minisart 0.45 μm Luer Lock Syringe Filter Cat no. FIL6588, Scientific Laboratory Supplies, United Kingdom). This stock solution was prepared fresh on the day of intranasal administration and was defined as 100% CSE.

Based on the median number of cigarettes smoked per day by the average United Kingdom smoker (healthsurvey.hscic.gov.uk), two cigarette smoke extract (CSE) concentrations were generated (See Appendix A). Stock solution was diluted to concentrations of either 1% (reflecting a light smoker consuming 5 cigarettes per day) or 3% (reflecting a heavy smoker consuming 15 cigarettes per day). These two CSE concentrations were chosen to be representative of experimentally defined lower- and higher-exposure conditions. Concentrations were selected based on a previously established CSE exposure protocol (Bartlett et al., 2026). Briefly, the number of cigarettes smoked per day per gram of average human weight was calculated, then multiplied by the average weight of an 8-week-old mouse, and finally converted to a percentage. While this method does not account for factors such as metabolic and lung structure differences, it provides a justifiable reasoning behind the concentrations mice were exposed to enable the study of the effects of smoking on reproductive health.

$$no.\;cigarettes\;per\;day÷average\;human\;weight\;\left(kg\right)=cigarettes\;per\;kg$$

$$cigarettes\;per\;kg÷1000=cigarettes\;per\;gram\;\left(g\right)$$

$$cigarettes\;per\;g\times average\;weight\;of\;8\;week\;old\;mouse\;\left(g\right)=no.\;cigarettes\;per\;mouse\left(no.\;cigarettes\;per\;mouse\times 7\right)\times 100=\%\;of\;cigarette\;smoke\;extract\;required$$

### 2.2 Animal exposure

All animal procedures within this study were conducted in accordance with the United Kingdom Animals (Scientific Procedures) Act 1986 and approved under a United Kingdom Home Office Project Licence (PPL PP5215372). Procedures were performed by appropriately licensed personnel under a Personal Licence, and all work was approved by the University of Nottingham Animal Welfare and Ethical Review Body. Animal care and experimental design adhered to NC3Rs guidelines for refinement and welfare.

After the 12-week dosing regimen, all mice were culled via intraperitoneal overdose of sodium pentobarbital (Dolethal, 200 mg/kg). Death was confirmed via exsanguination by cutting the femoral artery as stipulated under the United Kingdom Animals (Scientific Procedures) Act 1986. For each animal, the right ovary or testis were weighted, snap-frozen and stored at −80° C, while the left ovary or testis was fixed for histological analysis.

This exploratory study had twelve animals in total, four of which were randomly assigned to each of the three experimental groups, namely: control, 1% CSE, and 3% CSE. The biological and statistical unit of analysis was the mouse. The same animal’s ovarian follicles or seminiferous tubules were regarded as subsamples rather than distinct biological replicates. As this was an exploratory transcriptome profiling study, an *a priori* power calculation was not carried out and the small sample size should be taken into account when evaluating statistical power and generalisability of the results.

### 2.3 RNA isolation

Ovarian and testicular tissue samples (∼25 mg) were homogenised using a TissueLyser II (Qiagen) in 600 µL Buffer RLT Plus supplemented with anti-foaming reagent DX (0.5% v/v). Samples were processed in pre-chilled 2 mL RNase-free tubes containing a 5 mm stainless steel bead at 25 Hz for 30 s, with intermittent cooling on ice until complete homogenisation. Total RNA was extracted using the RNeasy Mini Kit (Qiagen) following the manufacturer’s instructions. Lysates were centrifuged 300 x g for 5 min, mixed with an equal volume of 70% ethanol, and applied to an RNeasy spin. On-column DNase I digestion was performed for 15 min at room temperature. Columns were washed and the RNA eluted in 30 µL RNase-free water. RNA concentration and purity were assessed using a NanoDrop spectrophotometer (Thermo Fisher Scientific).

### 2.4 Tissue microarray

Testicular and ovarian RNA were diluted to 100 ng/μl using RNase-free water (Qiagen, United Kingdom) before RNA integrity assessment on a Bioanalyzer 2,100 platform (Agilent, United Kingdom). Samples with an RIN ≥7 was used for the analysis. The GeneChip™ WT PLUS Reagent Kit (ThermoFisher Scientific, United Kingdom) was used to prepare RNA samples for whole transcriptome expression analysis using GeneChip™ Whole Transcript (WT) Expression Arrays, according to the manufacturer’s instructions. First-strand cDNA was synthesized from extracted RNA using Reverse Transcriptase, followed by second-strand cDNA synthesis using DNA Polymerase and RNase H. Complementary RNA (cRNA) was synthesized by *in vitro* transcription of second-strand cDNA using T7 RNA polymerase and was purified. The purified ss-cDNA was enzymatically fragmented and labelled with terminal deoxynucleotidyl transferase (TdT) using the provided DNA Labelling Reagent following the manufacturer’s instructions before hybridization onto the Clariom™ S Assay Mouse GeneChip™ (ThermoFisher Scientific, United Kingdom). Testicular and ovarian microarray data were analysed using the Partek Genomics Suite analysis software to generate differentially expressed gene lists. Comparative analysis between the three smoking groups was performed using standard one-way ANOVAs. Dysregulated transcripts were classified based on a False Detection Rate (FDR) = 0.05, fold change < +/−1.1, and p ≤ 0.05. As no genes were identified as being significantly differentially expressed when the FDR was applied, a subsequent analysis was conducted utilising a less stringent set of parameters (FC < +/−1.1, P ≤ 0.05). The DAVID web server was used for gene ontology (GO) functional annotation and enrichment analysis of transcripts corresponding to differentially expressed genes (DEGswith a FDR of ≤0.05 cut-off applied to denote significant terms. Gene lists for each smoking comparison were subsequently analysed using the WEB-based GEneSeTAnaLysis Toolkit (WebGestalt) for predicting the biological processes and phenotype using the mammalian phenotype ontology functional database (http://www.webgestalt.org/), with a FDR ≤ 0.05. Testicular and ovarian transcriptomic data sets have been deposited in GEO (Accession number GSE338254).

### 2.5 Histological analysis of testis and ovarian tissues

Testicular and ovarian tissues were fixed overnight in 4% formaldehyde at 4 °C (Sigma, Poole, United Kingdom) before being processed into paraffin wax for histological analysis. Tissues were sectioned at 5 μm and stained with haematoxylin and eosin (H&E). Sections were imaged and analysed using a Zeiss Axioscan 7 microscopy system (Zeiss, Germany). Ovarian histological analysis was conducted using four animals per exposure group (n = 4). For ovarian analysis, one section at a comparable tissue depth was selected from each ovary. Five randomly selected, non-overlapping 200 × 200 μm regions of interest per section were assessed by an investigator blinded to exposure group. Follicles were identified morphologically and classified as primordial, primary, secondary, early antral, antral, or preovulatory according to established follicle counting and tissue processing criteria (Adeniran et al., 2021). The presence of a zona pellucida (ZP) was recorded separately. Each follicle within the selected fields was counted once, and counts were summarised for each animal. For testicular analysis, seminiferous tubule area was determined in ImageJ by tracing the tubule perimeter with the freehand selection tool. Twenty randomly selected regions per testicular tissue were analysed, and the mean seminiferous epithelium height and lumen diameter were calculated for each animal.

### 2.6 Statistical analysis

The biological unit was the individual mouse. Measurements taken from different tissue areas, seminiferous tubules, or follicles in the same animal were combined or otherwise handled as within-animal subsamples and were not regarded as separate observations. Ovarian and testicular data were assessed for normality with SPSS (version 28) and analysed using a One-way ANOVA for normally distributed data or a Kruskal–Wallis test for non-normally distributed data with appropriate *post hoc* test. Data on seminiferous tubule area distribution above or below the 90th/10th percentiles of control tubules was analysed using a Chi squared test. Significance was taken at P < 0.05.

## 3 Results

### 3.1 Effects of cigarette smoking on mouse ovary

To explore the impact of cigarette smoke exposure on the mouse ovary, we assessed follicular staging in sections taken from our heavy-, light- and non-smoker exposed females (Figure 1A). We observed no overall difference in number of follicles at each stage of development (Figure 1B).

**FIGURE 1:**
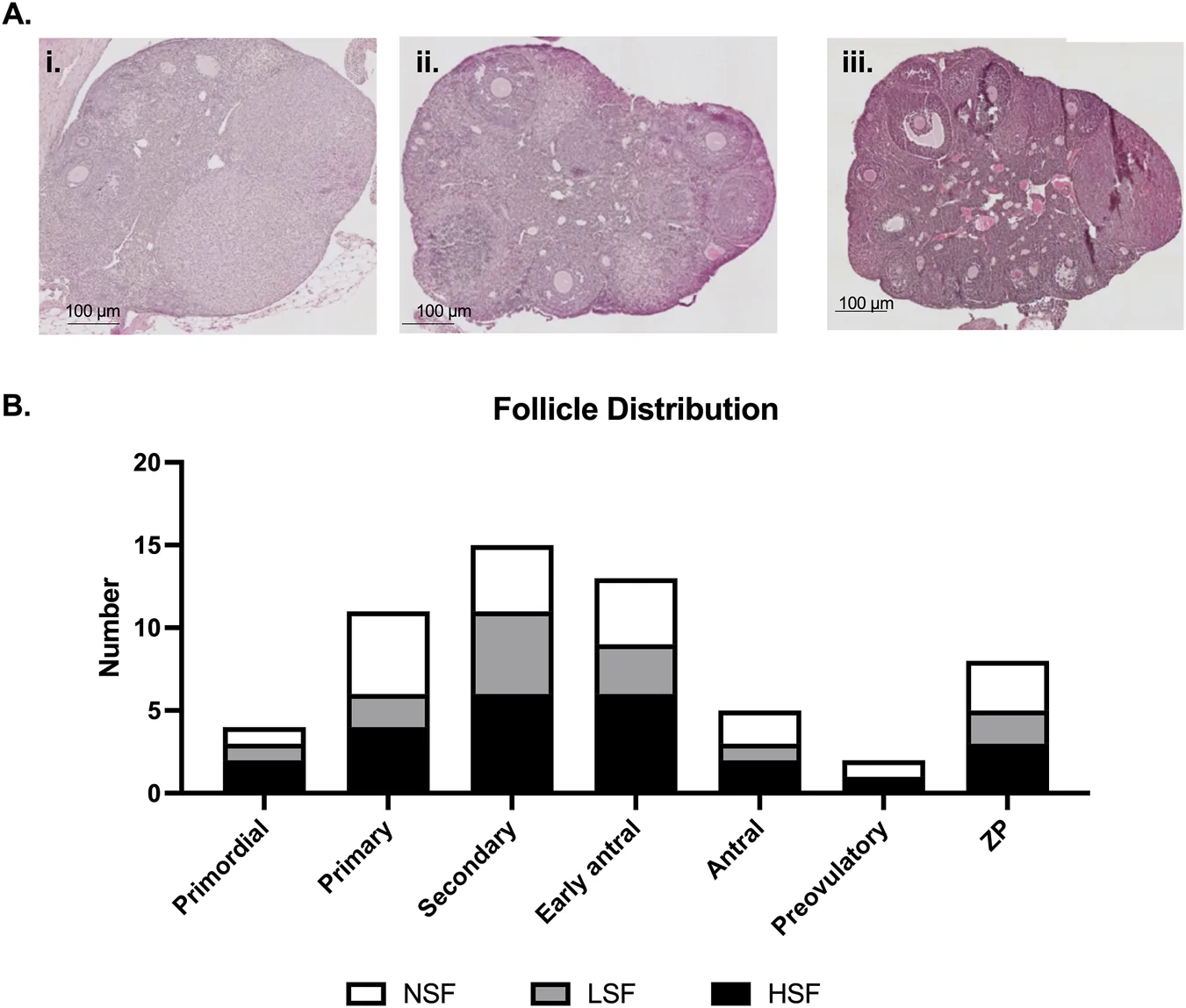
Effects of cigarette smoke extract exposure on ovarian follicular morphology. **(A)** Representative H&E-stained images of ovarian tissue sections taken from (i) non-smoker (ii) light-smoker and (iii) heavy-smoker females. **(B)** Ovarian follicular counts in non-smoker (NSF), light-smoker (LSF) and heavy-smoker females (HSF). N = 4 females per group. (ZP), Zona pellucida is a follicular structure and should not be interpreted it as a separate follicle stage.

Transcriptomic analysis of ovarian tissue from the same females identified differential expression of 1,010 genes (FC> +/− 1.1; P value < 0.05) between heavy-smoker and non-smoker females (Figure 2A), 1,683 genes between heavy-smoking and light-smoking females (Figure 2B) and 947 genes between light-smoking and non-smoking females (Figure 2C). Interestingly, comparison of overlapping differentially expressed genes between each group identified just 20 differentially expressed genes across all comparisons (Figure 2D).

**FIGURE 2:**
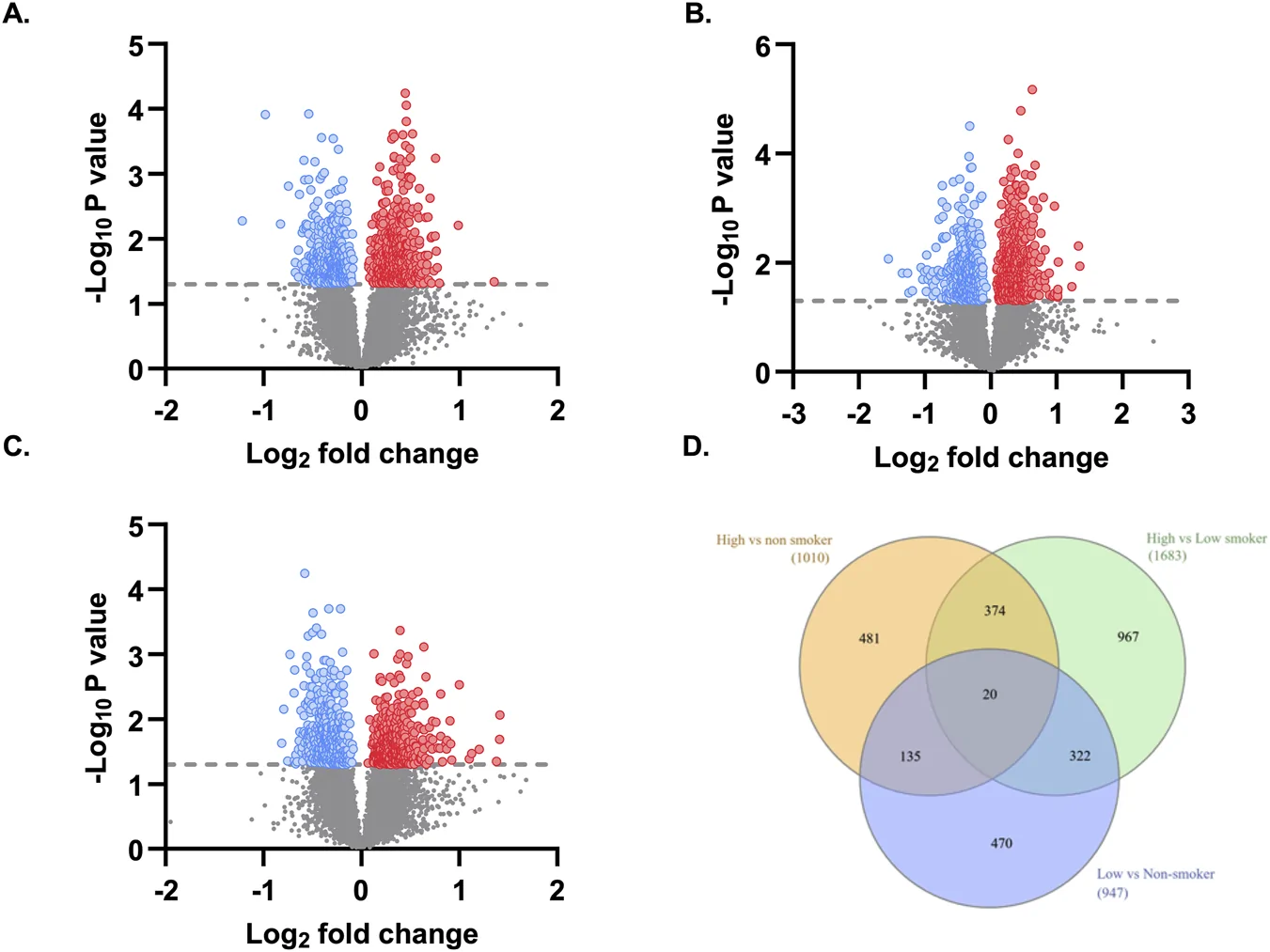
Impact of cigarette smoke exposure on ovarian transcriptomic profile. Volcano plots of differential gene expression between **(A)** heavy-smoker and non-smoker females, **(B)** heavy-smoker and light-smoker females and **(C)** light-smoker and non-smoker females. Venn diagram showing the differential (FC>±1.1, P < 0.05) gene expression between heavy-smoking, light-smoking and non-smoking females **(D)**. N = 4 ovarian samples per group.

Gene ontology analysis identified a downregulation of genes associated with cellular metabolism and transcription in ovaries from heavy-smoker females when compared to non-smoker females (Figure 3A). We observed a downregulated network (Figure 3B) of ubiquitin-mediated processes (Ubd, Cul1, Psmd1), epigenetic regulation (Setd1b, Setdb1, Kdm4d) multiple transcription factors (Stat2, Stat3, Rxra, Sox2) and several zinc finger proteins and DNA repair and RNA processing (Xrcc6, Smg1, Exosc1). Interestingly, the expression of multiple genes associated with cytoplasmic translation were also increased in the ovaries from heavy-smoker females when compared with non-smoker females (Figure 3C). Here, we observed increased expression of multiple ribosomal proteins (Figure 3D) as well as genes linked to ovarian cancer (Wnt5a, Tlr3, Lmnb1, Tnfsf11, Tex19.1). Next, we compared the transcriptional profile of ovarian tissue from heavy-smoking females with that from non-smoker females. Here, we observed a downregulation of genes associated with non-coding RNA (ncRNA) and metabolic processes in heavy-smoking females (Figure 3E). Several of these genes were the same as observed when comparing the heavy-smoker females to the non-smoker females (e.g. Stedb1, Xrcc6, Kdm4d). However, we also observed a downregulation of multiple genes associated with RNA methylation (Mettl2, 14, 15 and 16) and RNA processing (Exosc1/9) (Figure 3F). Analysis of upregulated genes identified an enrichment associated with translation regulation (Figure 3G), related to the increased expression of multiple ribosomal genes (Figure 3H) in heavy-smoker females. Finally, we compared the expression profile of ovarian tissue from light-smoker females with non-smoker females. We observed a downregulation of macromolecular metabolism linked to transcriptional regulation (Figure 3I) in light-smoking females. Here, we observed downregulation of multiple elongation factors (Eef2, Eef2k) (Figure 3J), transcription factors (Foxg1, Foxl2, Foxs1, Gli2, Rara, Rxra), and epigenetic regulators (Sirt7, Dnmt3b, Kat5). Analysis of upregulated genes identified an enrichment for genes related to cell death, DNA damage and inflammation (Figure 3K), including Il-6, Ptgs2, Tlr3/5 and Hus1 (Figure 3L).

**FIGURE 3:**
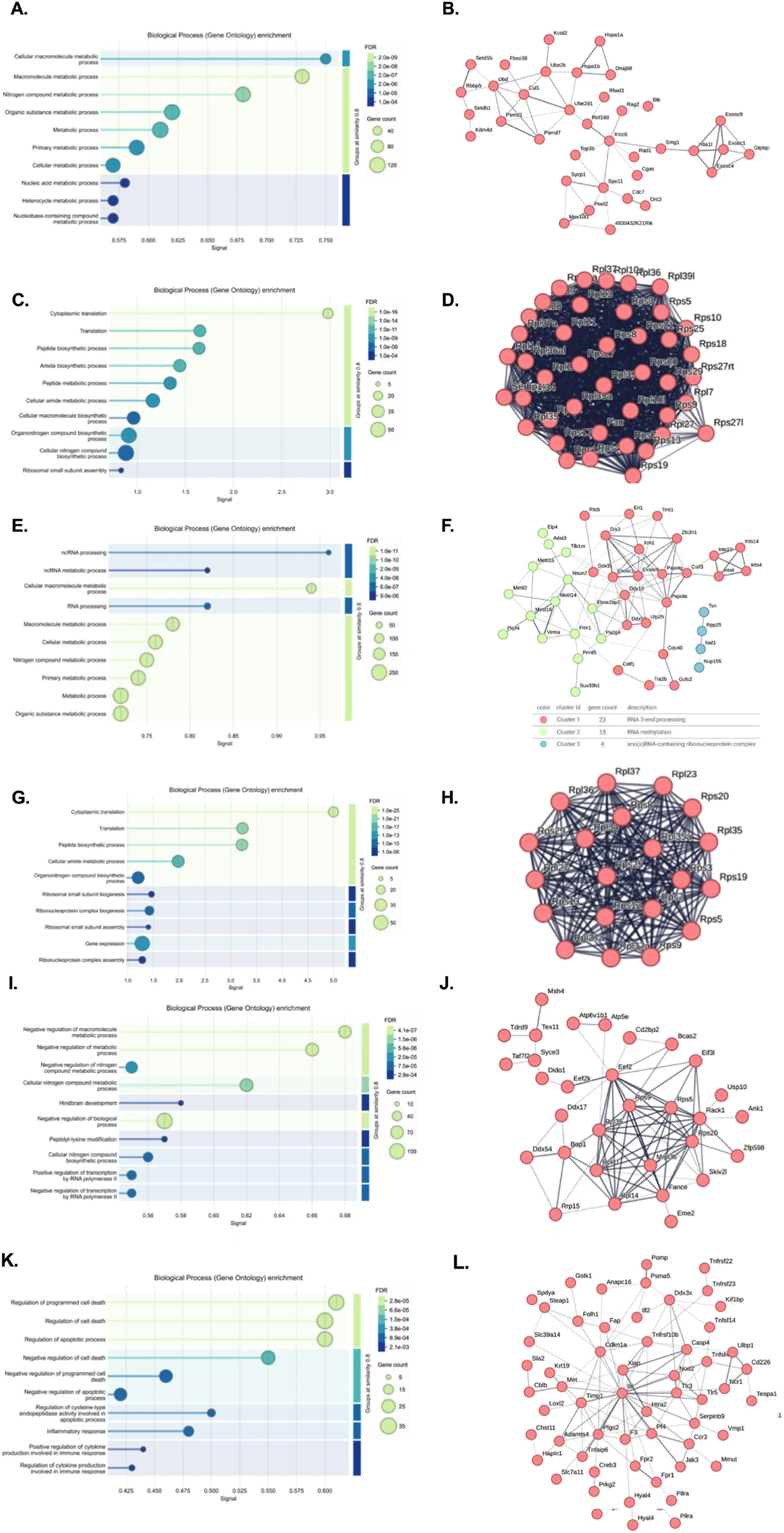
Gene ontology analysis of genes. Gene ontology (biological processes) **(A,C)** and network analysis of upregulated and downregulated genes in heavy-smoking vs. non-smoking ovarian tissues respectively **(B,D)**. Gene ontology (biological processes) **(E,G)** and network analysis of upregulated and downregulated genes in heavy-smoker vs. light-smoker ovarian tissues, respectively **(F,H)**. Gene ontology (biological processes) **(I,K)** and network analysis of upregulated and downregulated genes in light-smoker vs. non-smoker ovarian tissues, respectively **(J,L)**. N = 4 ovarian samples per group.

### 3.2 Effects of cigarette smoking on mouse testis

To explore the impact of cigarette smoke exposure on the mouse testis, we undertook histological analysis of H&E-stained testicular tissue sections from heavy-smoker (Figure 4Aiii), light-smoker (Figure 4Aii) and non-smoker (Figure 4Ai).

**FIGURE 4:**
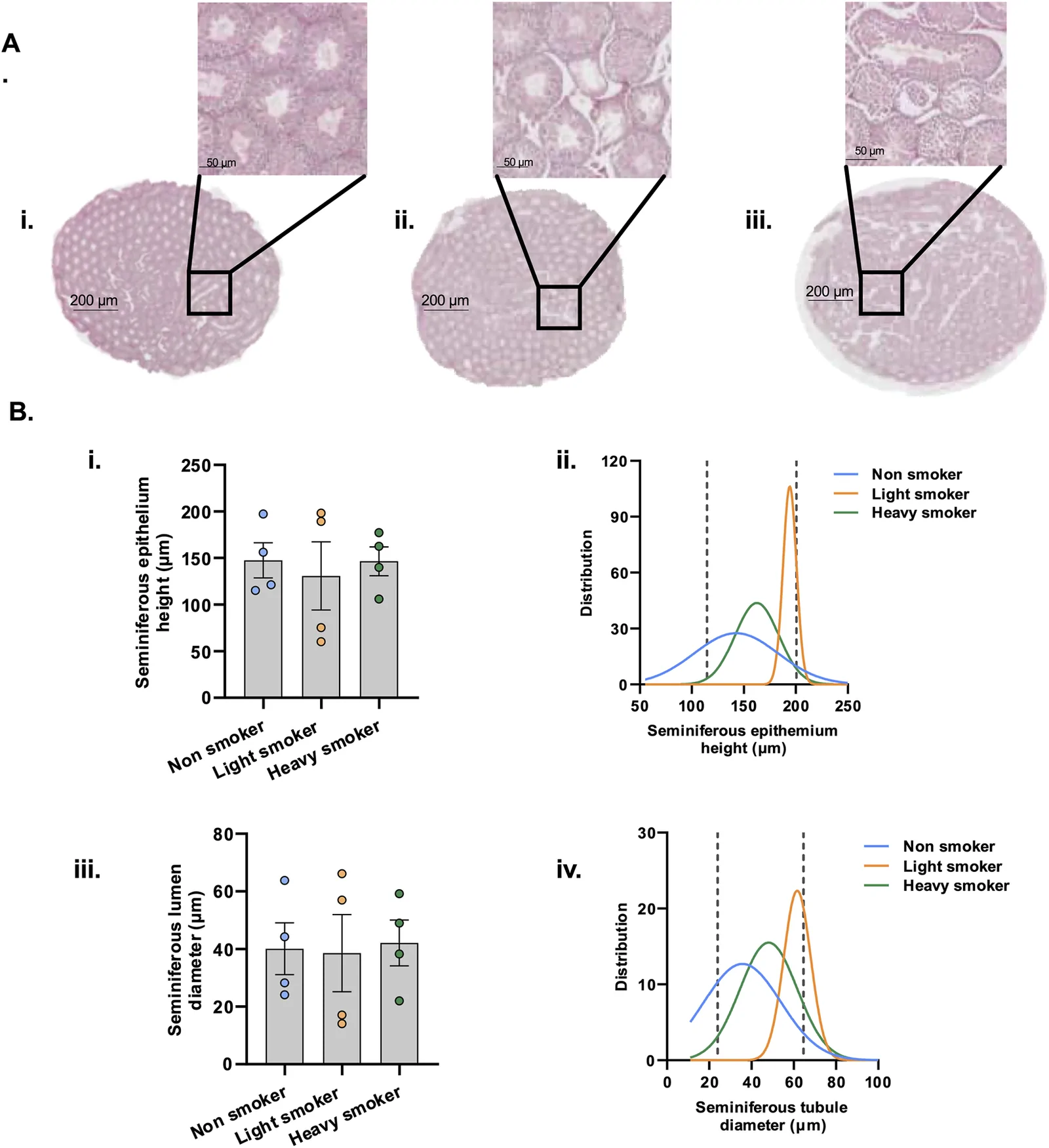
Effects of cigarette smoke extract exposure on testicular morphology. **(A)** Representative H&E-stained images of testicular tissue sections taken from (i) non-smoker (ii) light-smoker and (iii) heavy-smoker males. **(B)** Testicular counts of seminiferous epithelium height and lumen diameter from non-smoker, light-smoker and heavy-smoker males. N = 4 males per group. A chi-square analysis was conducted to examine the association between groups.

While we observed no difference in the mean height of the seminiferous tubule epithelium across groups (Figure 4Bii), analysis of tubule size distribution revealed light smoker males had a significantly higher proportion of tubules which were above the 90th percentile area of the non smoker males (P < 0.0001), (Figure 4Bii). Conversely, in the heavy smoker males, there were an increased number of tubules that fell below the 10th percentile of the control males for tubule epithelial height (P = 0.0004), (Figure 4Bii). Similarly, analysis of the tubule lumen diameter revealed no mean difference between groups (Figure 4Biii). However, both the light- and heavy smokers displayed a lesser number of tubules with lumen diameters below the 10th percentile of the control males, while the heavy smokers had a higher number of tubules with lumen diameters above the 90th percentile of the controls (all P < 0.0001), (Figure 4Biv).

Finally, we analysed the transcriptomic profile of testicular tissue collected from our heavy-smoker, light-smoker and non-smoker males. We identified differential expression of 1,641 genes (FC> +/− 1.1; P value < 0.05) between heavy-smoker and non-smoker males (Figure 5A), 1,498 genes between heavy-smoker and light-smoker males (Figure 5B) and 788 genes between light-smoker and non-smoker males (Figure 5C). Interestingly, unlike in our females, comparison of overlapping differentially expressed genes between each group identified 200 differentially expressed genes across all comparisons (Figure 5D).

**FIGURE 5:**
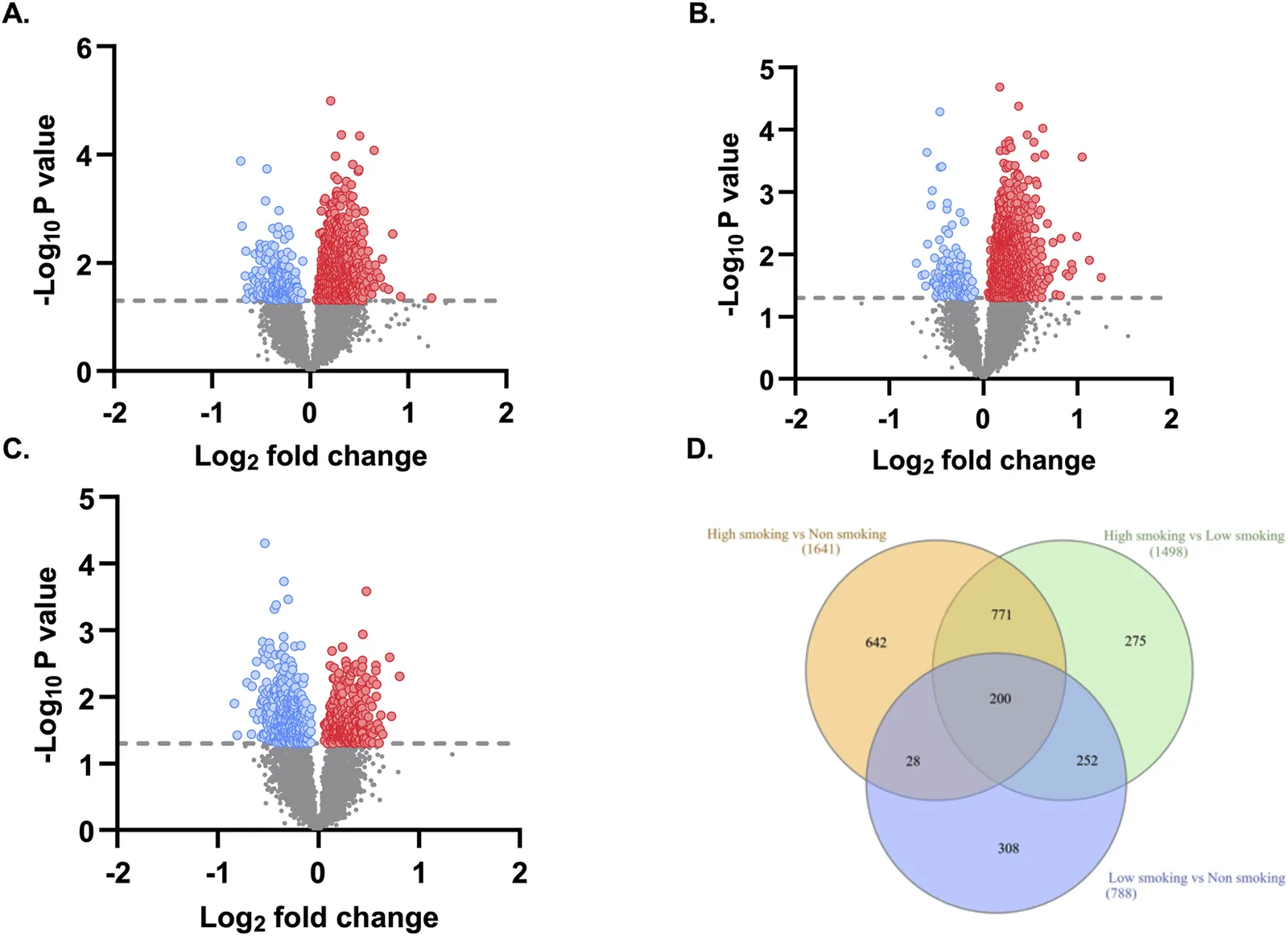
Effects of cigarette smoke extract exposure on testicular transcriptional profile. Volcano plots of differential gene expression between **(A)** heavy-smoking and non-smoking males, **(B)** heavy-smoking and light-smoking males and **(C)** light-smoking and non-smoking males. Venn diagram showing the differential (FC>±1.1, P < 0.05) gene expression between heavy-smoking, light-smoking and non-smoking males **(D)**.

Gene ontology analysis identified a downregulation of genes associated with tyrosine phosphorylation, leukocyte activation and Jak/Stat signalling in testes from heavy-smoker males when compared to non-smoker males (Figure 6A). We observed a downregulated network comprising central cytokine (Csf2, Socs2), inflammatory (Il9) and immunological (Ctsg) regulators (Figure 6B). When assessing genes upregulated in heavy-smoking males, we observed enrichment of gene associated with spermatogenesis and male reproduction (Figure 6C). Within these, genes central for Sertoli cell function (Sox9), cellular proliferation (Kit), spermatid maturation (Spem1) and epigenetic regulation (Piwl1) were all increased (Figure 6D). Comparing the transcriptional profile in testicular tissue from heavy-smoker and light-smoker males identified a downregulation of multiple olfactory receptors (Figure 6E) however, no significant networks were identified (Figure 6F). In contrast, we observed significant upregulation of multiple genes associated with immune system regulation (Figure 6G), associated with T-cell activation (Ptprc), immune response (Irf1, Il7r, Il23r, Fcgr1, Rfer1g) and cellular proliferation (Kit) (Figure 6H). Finally, comparison of tissue from light-smoker males with non-smoker males identified a downregulation of genes involved in epithelial function, gene expression regulation and immune responses (Figure 6I). Here, we observed a network comprising multiple regulators of T-cell activation (Ptprc), cell-cell connection (Itgb2, Itga4, Icam1) and immune responses (Ctsg, Cd27, Csf1) (Figure 6J). Analysis of upregulated genes showed a diverse profile of genes with enrichment for histone binding and olfactory receptors (Figure 6K). However, network analysis showed these genes to have minimal interaction with each other (Figure 6L).

**FIGURE 6:**
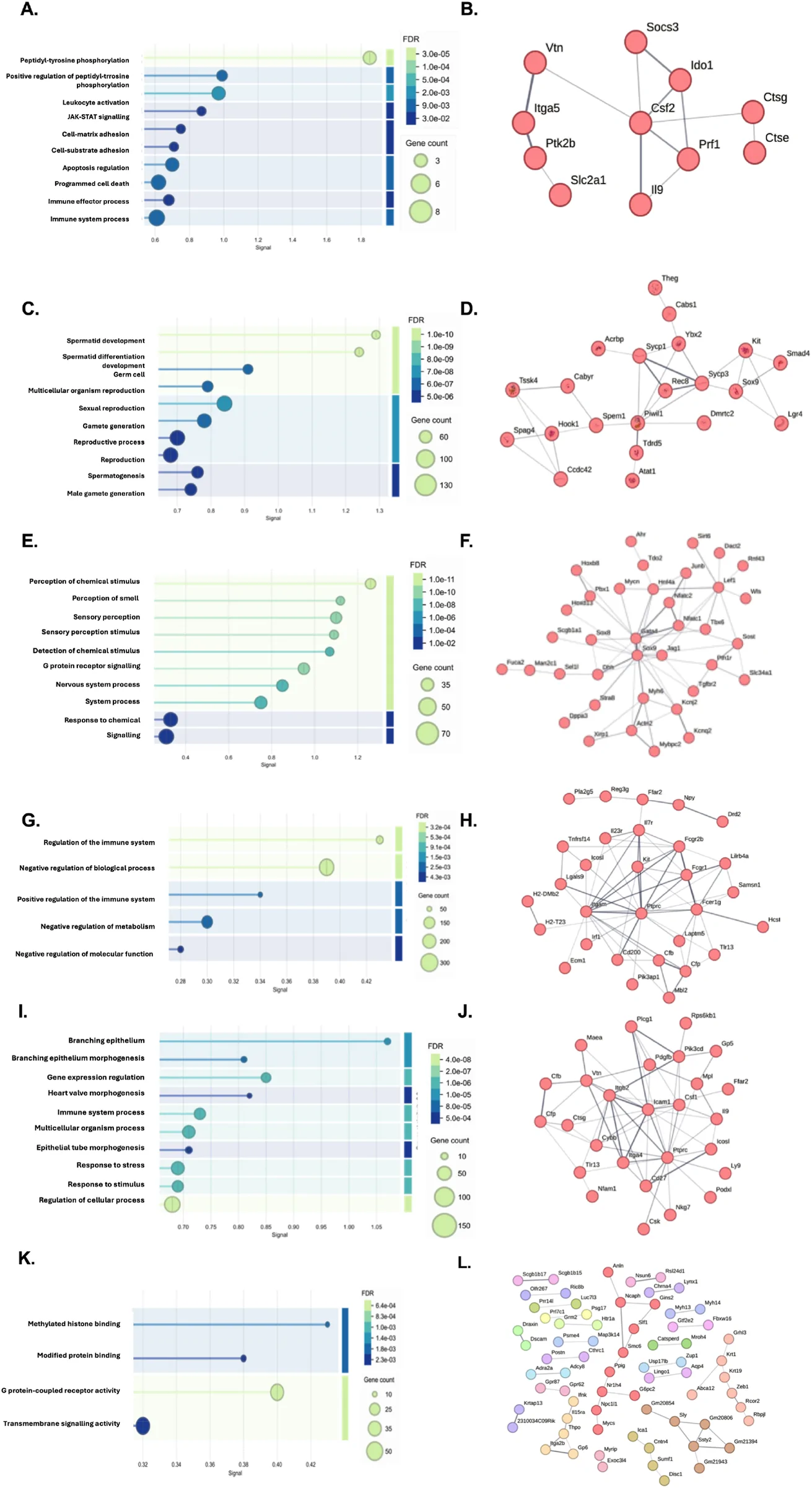
Gene ontology analysis of genes. Gene ontology (biological processes) **(A,C)** and network analysis of upregulated and downregulated genes in heavy-smoking vs. non-smoking testicular tissues, respectively **(B,D)**. Gene ontology (biological processes) **(E,G)** and network analysis of upregulated and downregulated genes in heavy-smoker vs. light-smoker testicular tissues, respectively **(F,H)**. Gene ontology (biological processes) **(I,K)** and network analysis of upregulated and downregulated genes in light-smoker vs. non-smoker testicular tissues, respectively **(J,L)**. N = 4 testicular samples per group.

## 4 Discussion

The detrimental effects of cigarette smoking on reproductive health are well-established (Dechanet et al., 2011) (Simpson, 1957). Smoking remains a major modifiable public health concern, contributing heavily to fertility issues, gestational complications and poor birth outcomes (Bhardwaj et al., 2025). However, the underlying molecular and cellular mechanisms linking cigarette smoke exposure with poor reproductive health have yet to be fully defined. We have established a refined model in which male and female mice are exposed to an intranasal cigarette smoke extract over several weeks. In the present study, we used this model to assess the impact of cigarette smoke exposure on male and female reproductive health. We observed minimal impact of our cigarette smoke extract on ovarian morphology and follicle number. In contrast, seminiferous tubules from males exposed to the highest concentration showed significant enlargement. Transcriptomic profiling revealed perturbed regulation of metabolic, translation, RNA processing, and cell death genes in ovaries. Conversely, testicular tissues showed altered expression of genes regulating spermatogenesis, immune responses, histone binding, and signal transduction.

Globally, smoking prevalence among women of reproductive age (typically 15–49 years) averages 15%–20% (Jackson et al., 2024). Previous studies have shown that smoking causes deleterious effects on the ovary, disrupting the secretion and abundance of sex steroid hormones (Neal et al., 2007; Soldin et al., 2011). As the toxic components of cigarette smoke bioaccumulate in follicular fluid (Paccola et al., 2021), the toxic effects of smoking might be seen more in the ovary than in other tissues. However, we observed that exposure to either a 1% (light-smoking group) or 3% (heavy-smoker group) cigarette smoke extract has minimal impact on ovarian morphology and follicle counts. Interestingly, a recent study in rats showed that maternal exposure to 2 mg/kg/day of nicotine did not affect female fertility, as measured by the number of oocytes released, but did affect oocyte morphology (Paccola et al., 2021). Other studies have also reported no overall change in the number of oocytes retrieved from adult mice exposed to nicotine was observed (Jennings et al., 2011). However, increased thickness of the zona pellucida, higher rates of meiotic spindle abnormalities (Jennings et al., 2011; Shiloh et al., 2004) and delayed rates of follicle maturation were reported (Neal et al., 2007). Together with our data, these studies suggest that while fundamental female fertility might not be altered, follicle maturation and chromosomal abnormalities might be increased. However, more detailed analyses of ovarian cell types, oocyte morphology and chromosomal integrity are needed to fully define how maternal cigarette smoke exposure affects ovarian function.

Assessing the transcriptomic profile in our heavy-smoking ovaries, we observed significant downregulation of multiple transcription regulation, epigenetic maintenance and DNA damage genes including central transcription factors (Sox2, Stat2, Rxra and Gli2). In the ovary, Sox2 has mainly been explored in regard to its role in promoting ovarian cancer where it helps ovarian cancer cells retain stem-cell-like properties, enabling them to migrate, and invade surrounding tissues. (Wen et al., 2017). Similarly, overexpression of Stat2, Rxra and Gli2 have also been associated with ovarian cancer, cell proliferation and immune responses stimuli (Lee et al., 2020; Chen et al., 2022; Wang et al., 2021). Therefore, these observations could potentially indicate a coordinated downregulation of cell division regulation. Within the ovary, one of the main proliferating cell types is the granulosa cell. Granulosa cells have multiple roles including supporting the maturation of the oocyte and production of oestrogen. Extrinsically, proliferation of granulosa cells is regulated by levels of Follicle-Stimulating Hormone (FSH) and estrogen, alongside local growth factors like activin and TGF-β. Intrinsically, cyclins are key regulatory proteins that drive cell cycle progression, with Cyclin D2 acting as the master regulator of granulosa cell proliferation. However, as we did not measure levels of circulating hormones, and the expression of key regulators such as Gdf9 and Bmp15 were unaltered, we are unable to determine whether granulosa cell proliferation was downregulated in our high-smoking females.

Analysis of genes upregulated in our heavy-smoker females revealed differential ex-pression of multiple ribosomal proteins. Ribosomal proteins such as Rplp1 are critical factors for protein synthesis and cell proliferation. Here, the upregulation of multiple ribosomal proteins contrasts that of the downregulated transcription factors in the same females, indicating elevated protein synthesis but in the absence of cell division, as occurs within the post-mitotic maturing oocytes (Sanchez et al., 2016). Interestingly, increased expression of ovarian ribosomal proteins has been linked to Premature Ovarian Insufficiency (POI). This upregulation is seen as a compensatory response to mitochondrial dysfunction and oxidative stress, which ultimately drives premature follicle loss and ovarian aging (Shi et al., 2023). In POI, the resting primordial follicle pool is exhausted, and ovarian granulosa cell proliferation is severely downregulated (Zhang et al., 2022). As smoking in women is associated with early natural menopause (Whitcomb et al., 2018), our model could be useful for understanding the molecular mechanisms linking environmental factors with declining female fertility. However, as we did not measure levels of circulating hormones, ovulation rates or markers of oocyte quality in our mice, the potential impacts on female fertility are yet to be determined.

Comparison of ovarian tissue from our light-smoking females with our non-smoking females showed a similar profile with a downregulation of multiple transcription factors (Gli2, Tead2, Meox1, Rara, Rxra, Stat2) and epigenetic regulators (Sirt7, Kat5, Setd1b, Setd5, kmt2d) and an upregulation of multiple ribosomal proteins. Females exposed to the lower concentration of cigarette smoke also displayed an ovarian transcriptional profile indicating reduced proliferation and increased protein synthesis, indicative of POI. Studies in women have indicated a dose-dependent effect of smoking on menopause (Whitcomb et al., 2018), with women who quit smoking in their 40s versus their 30s having a higher menopause risk than women who quit in their 20s.

Our second focus centred on male reproductive health. Comparing testicular histology across groups, we observed an increased mean tubule cross section area in the heavy-smoker males. Increases in tubule cross section area can be caused by increased cell proliferation or the buildup of testicular fluid due to efferent duct blockage (Jeffs et al., 2001; Nakata et al., 2026). In men, a lack of sperm in the ejaculate, known as azoospermia, accounts for 10%–15% of male infertility cases (Minhas et al., 2021). At present, smoking has not been linked with obstruction of the rete testis of efferent ducts. However, smoking has been shown to contribute to azoospermia and rete testis issues primarily by elevating oxidative stress and DNA damage within the testicles (Bundhun et al., 2019). Similar to the ovary, our analysis of the testis only explored gross morphology. Therefore more detailed investigation of testicular cell populations is required. Additionally, assessment of testicular cell proliferation/apoptosis, antioxidant status and stress responses, would provide further insight into the cellular responses to cigarette smoke extract exposure.

Analysis of testicular transcriptomic profiles suggested a modest downregulation of genes associated with immune modulation in males exposed to the highest concentration of cigarette smoke extract. Within the seminiferous tubules, Sertoli cells are the testes’ primary immune regulators, creating an immunosuppressive environment that prevents systemic immune cells from destroying immunogenic sperm, while carefully maintaining innate defences against infection (Bhardwaj et al., 2025). The downregulation of immune processes within our heavy-smoking males could potentially indicate an impairment in normal Sertoli cell function, however, additional studies are needed to confirm this. In contrast, we observed significant upregulation of multiple genes central to spermatogenesis and transcription regulation in our heavy-smoker males. Here, genes such as Brdt, Spem1, Piwil1 and Sox9 were all increased. Brdt (Bromodomain Testis-associated) is a testis-specific protein responsible for regulating chromatin organization and gene expression during sperm development. During the later stage of spermatogenesis, core histones are hyperacetylated and then replaced by transition proteins prior to protamines (Barral et al., 2017). As such, histone acetylation forms an essential function in regulating spermatogenesis and testicular gene expression, with its impairment linked to male infertility (Kohandani et al., 2022). Similar to Brdt, PIWIL1 is expressed in germline cells within the testes, particularly in pachytene spermatocytes and spermatids. Piwil1 binds to PIWI-interacting RNAs (piRNAs) to protect the genome, regulate gene expression, and guide the translation of post-meiotic mRNAs required for sperm formation (Wu et al., 2018). However, when overexpressed, PIWIL1 drives tumor cell proliferation (Dong et al., 2021). Spem1 also regulates later stages of spermatogenesis through the removal of excess cytoplasm. Impairment in this process results in the production of abnormal sperm displaying structural deformities such as being bent or coiled (Zheng et al., 2007). Separate to the germ cells of the testis, Sox9 is a master regulator of Sertoli cell function and integrity. As Sertoli cells are the nurse cells of the seminiferous tubules, their function is essential for spermatogenesis. Interestingly, elevated SOX9 expression has been observed in men with Non-Obstructive Azoospermia, where a lack of germ cells within the testis results in Sertoli Cell Only Syndrome (Ghanami et al., 2021). As noted earlier, more detailed analyses of the testes, as well as circulating hormone profiles, of our males are needed to understand how smoking affects testicular, spermatogenesis and sperm epigenetic status.

When comparing our light-smoker males to the non-smoker males, we observed a downregulation of multiple cell signaling and transcription regulating genes such as Sox8/9, Nkx3-1, Gata4 and Dhh. As discussed already, Sox8 and Sox9 are fundamental regulators of Sertoli cell function, maintain the integrity of the blood testis barrier and spermatogenesis (Chalmel et al., 2013). Nkx3-1 is also expressed in the Sertoli cells as well as the spermatogonial stem cells and is downregulated in highly invasive cancers like seminomas and embryonal carcinomas (Skotheim et al., 2003). Similarly, Gata4 is primarily expressed in the somatic (Sertoli cells and Leydig cells) where it regulates gene expression essential for sex steroid production, sperm maturation, and the maintenance of the blood-testis barrier, while Dhh is also critical for the normal structure and function of the seminiferous tubule (Min et al., 2022). These data suggest regulators of testicular and Sertoli cell function could be perturbed by exposure to cigarette smoke. However, additional functional studies are required to confirm this.

Finally, we observed an upregulation of genes known to be associated with immune cell responses, including Ptprc, Kit, Itgam and Irf1. Protein tyrosine phosphatase receptor type C (Ptprc), also known as CD45, and Integrin Subunit Alpha M (Itgam), are both cell surface markers used to identify white blood cells (leukocytes). Normal testicular tissue generally contains only a very small percentage (roughly 5%) of CD45 and Itgam-positive immune cells, which serve to protect from autoimmune attacks while supporting healthy tissue function. High levels of CD45 and Itgam positive cells typically point to underlying infections, orchitis, or inflammation within the male reproductive tract, which can negatively impact sperm quality and male fertility (Sharma et al., 2022). Testicular Mast/stem cell growth factor receptor (known as KIT or c-KIT) is a cell-surface tyrosine kinase receptor essential for spermatogenesis, germ cell survival, and mast cell development, regulating the proliferation and maintenance of early-stage germ cells (Chalmel et al., 2013; Cardoso et al., 2017). Together, these data might indicate that males exposed to the light-smoking regimen could have a pro-inflammatory response within their testes, mediated potentially by an elevated presence of leukocytes. However, more detailed analyses of the testes from our males are required to confirm these hypotheses.

To conclude, using our model, we observed reproductive tissue-specific responses in the ovary and testis while having no discernible impact on animal welfare. Within the ovary, we observed a transcriptomic profile suggestive of reduced cellular proliferation, coupled with increased protein translation. In contrast, testicular tissue showed differential responses to the heavy- and light-smoking doses, with associated changes in testicular structure and differential expression of genes involved in spermatogenesis regulation. Our study is limited by the low number of animals assessed and by the lack of validation of the transcriptomic and morphological changes observed. Therefore, additional studies are needed to understand how the tissue-specific changes reported here translate into reproductive outcomes for our males and females, and their offspring. Despite the preliminary nature of this study, we believe our model provides new insight into how smoking may contribute to impaired fertility and reproductive health in men and women.

## Data Availability

The testicular and ovarian transcriptomic data sets presented in the study are publicly available. This data can be found in the GEO repository with the accession number GSE338254.
