# Supplementary material for: Cigarette smoke extract exposure induces differential morphological and transcriptional responses in the ovary and testes of mice
Source: Front Toxicol. 2026 Oct 14;8:1943043. doi: 10.3389/ftox.2026.1943043 (PMC13713111; doi:10.3389/ftox.2026.1943043)
Supplement: Supplementary file 1 [file Supplementaryfile1.docx]

Appendix A

**Figure 1 – Generation of cigarette smoke extract (CSE).** A custom smoke-collection apparatus consisting of plastic piping connected to a three-way stopcock was used. The stopcock was attached to one side to a 50 mL BD Plastipak polypropylene disposable Luer-Lok concentric-tip, while the other was connected to a modified 25 mL tube lid, allowing attachment of the CSE collection tube. Marlboro Red cigarettes were used as the standardised cigarette and were secured using 1 mL pipette tips with the conical ends removed. The cigarette smoke was drawn through phenol-red–free RPMI-1640 culture medium, prior to sterile filtration using a 0.45 μm. This stock solution was prepared fresh on the day of intranasal administration and was defined as 100% CSE.

**Calculations for exposure:**

Calculations were made to determine the percentage of CSE to be used. This was performed by calculating the number of cigarettes smoked per day per gram of average human weight, and calculating how many cigarettes smoked per week per average weight of an 8-week-old mouse in percentage form. While this method does not account for factors such as metabolic and lung structure differences, this method provides a justifiable reasoning behind the concentrations mice were exposed to. As such, the following calculations were performed to determine CSE doses:

$$no. cigarettes per day \div average human weight \left( kg \right) =cigarettes per kg$$

$$cigarettes per kg \div1000 =cigarettes per gram \left( g \right)$$

$$cigarettes per g \times average weight of 8 week old mouse \left( g \right)=no. cigarettes per mouse$$

$$\left( no. cigarettes per mouse \times7 \right)\times100 = \% of cigarette smoke extract required$$

$$Light Smoker Males:$$

$$5 \div84.5 =0.05855 cigarettes per kg$$

$$0.05855 \div1000 =0.00005855 cigarettes per g$$

$$0.00005855 \times25=0.001464$$

$$\left( 0.001464 \times7 \right)\times100 = 1.02 \%$$

$$Light Smoker Females:$$

$$5 \div72.1 =0.06935 cigarettes per kg$$

$$0.06935 \div1000 =0.00006935 cigarettes per g$$

$$0.00006395 \times20=0.001387$$

$$\left( 0.001387 \times7 \right)\times100 = 0.97 \%$$

$$Heavy Smoker Males:$$

$$15 \div84.5 =0.17564 cigarettes per kg$$

$$0.17564 \div1000 =0.00017564 cigarettes per g$$

$$0.00017564 \times25=0.004391$$

$$\left( 0.004391 \times7 \right)\times100 = 3.07 \%$$

$$Heavy Smoker Females:$$

$$15 \div72.1 =0.20804 cigarettes per kg$$

$$0.20804 \div1000 =0.00020804 cigarettes per g$$

$$0.00020804 \times20=0.0041608$$

$$\left( 0.0041608 \times7 \right)\times100 = 2.91 \%$$
